# Ophiuroids Discovered in the Middle Triassic Hypersaline Environment

**DOI:** 10.1371/journal.pone.0049798

**Published:** 2012-11-19

**Authors:** Mariusz A. Salamon, Robert Niedźwiedzki, Rafał Lach, Tomasz Brachaniec, Przemysław Gorzelak

**Affiliations:** 1 Department of Palaeontology and Biostratigraphy, University of Silesia, Faculty of Earth Sciences, Sosnowiec, Poland; 2 Institute of Geological Sciences, Wroclaw University, Wroclaw, Poland; 3 Department of Geochemistry, Mineralogy, and Petrography, University of Silesia, Sosnowiec, Poland; 4 Department of Biogeology, Institute of Paleobiology, Polish Academy of Sciences, Warsaw, Poland; The Australian National University, Australia

## Abstract

Echinoderms have long been considered to be one of the animal phyla that is strictly marine. However, there is growing evidence that some recent species may live in either brackish or hypersaline environments. Surprisingly, discoveries of fossil echinoderms in non-(open)marine paleoenvironments are lacking. In Wojkowice Quarry (Southern Poland), sediments of lowermost part of the Middle Triassic are exposed. In limestone layer with cellular structures and pseudomorphs after gypsum, two dense accumulations of articulated ophiuroids (*Aspiduriella similis* (Eck)) were documented. The sediments with ophiuroids were formed in environment of increased salinity waters as suggested by paleontological, sedimentological, petrographical and geochemical data. Discovery of Triassic hypersaline ophiuroids invalidates the paleontological assumption that fossil echinoderms are indicators of fully marine conditions. Thus caution needs to be taken when using fossil echinoderms in paleoenvironmental reconstructions.

## Introduction

Numerous studies dealing with salinity level and its impact on modern echinoderms showed that this parameter is important in terms of their spatial distribution and size [1]–[8]. In general, modern echinoderms have a more limited salinity range than any other invertebrates because they have a permeable body wall and lack differentiated osmoregulatory or excretory organs [2]–[9]. This limit can especially be drawn for fossil echinoderms, among which only stenohaline taxa have been documented. Although, some authors recently described Messinian echinoids from non-normal saline deposits deposited during evaporitic episode of the Mediterranean region [10], [11]; however, it seems that these echinoids [*Brissopsis* gr. *lyrifera* (Forbes)] occurred in interbedded horizons with rather normal(?) salinity (personal communication, A. Kroh, Naturhistorisches Museum Wien).

Intriguingly, *in situ* and labolatory experiments demonstrated that some extant echinoderms, in particular ophiuroids, asteroids and holothurians, can tolerate a great range of salinities [12]. So far, an ophiuroid *Ophiophragmus filograneus* (Lyman) constitutes an echinoderm species living in the lowest salinity in the field [13]. This form was found in the brackish facies of salinity level of 7.7%. Remarkable salinity tolerance of this species has been also confirmed under laboratory conditions [14], [15]. Likewise, many authors draw attention to the fact that certain species of echinoderms can tolerate very low salinity [7]–[16]. Additionally, it has been pointed out that low salinity may lead to size abnormalities (dwarfism) [17]–[20].

On the other hand, echinoderms can tolerate hypersaline conditions (i.e.,>35.5 psu according to [21]). For example, Price [22] reported two ophiuroid [*Amphipholis squamata* (Chiaje) and *Amphiura fasciata* Mortensen), and one holothurian (*Leptosynapta chela* Mortensen) species in Arabia Gulf with salinity ranging form 52 to 55%. Furthermore, the latter author documented two stunted asteroid species (*Astropecten polyacanthus phragmorus* Fisher and *Asterina burtowi* Gray) in lagoons with salinity exceeding 60% which to date constitute the highest record for salinity tolerance by echinoderms in the wild.

In this paper, we report for the first time well-preserved ophiuroids *Aspiduriella similis* (Eck) from the Middle Triassic sediments of Poland that were deposited in hypersaline conditions.

## Materials and Methods

### Geological Setting

**Figure 1:**
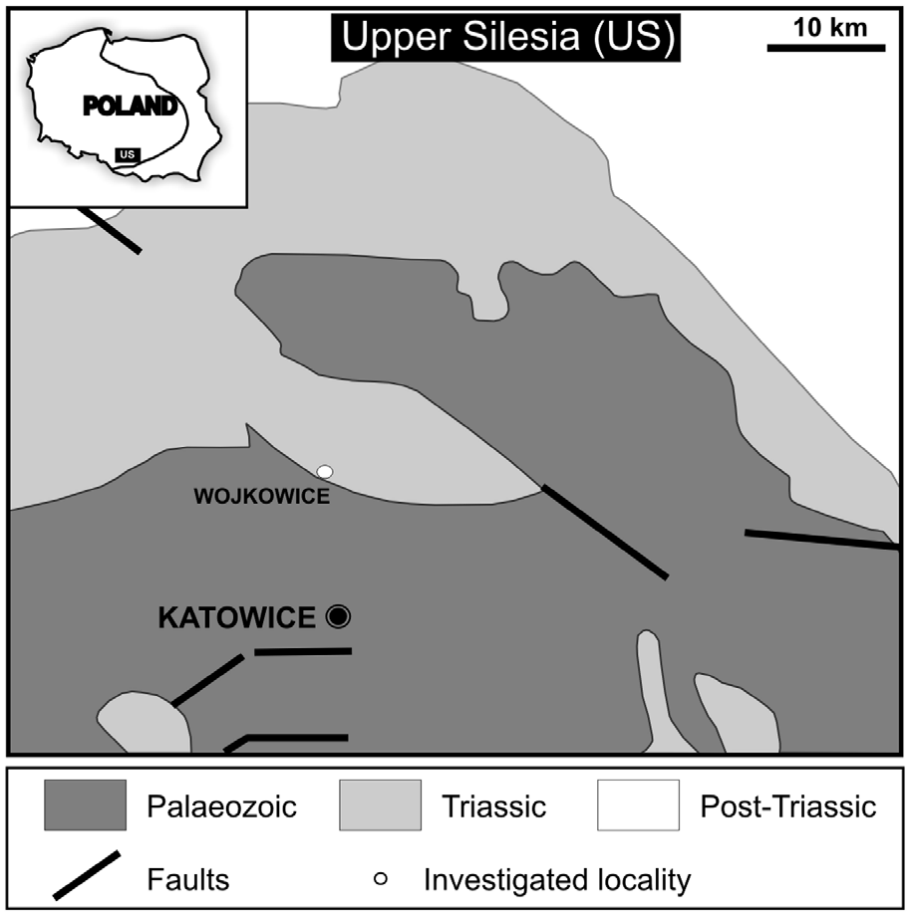
Fossil locality and geological setting. Map of Poland with investigated area indicated and enlargement of Upper Silesia with the sampled Wojkowice Quarry (circle). Figure slightly modified from [25].

Abandoned quarry “Wojkowice” is situated in the so-called Silesian-Cracow Monocline, in Upper Silesia, Southern Poland (Figure 1). This monocline contains mainly Triassic sediments deposited in the Germanic Basin on the northern margin of the Tethys Ocean [23].

**Figure 2:**
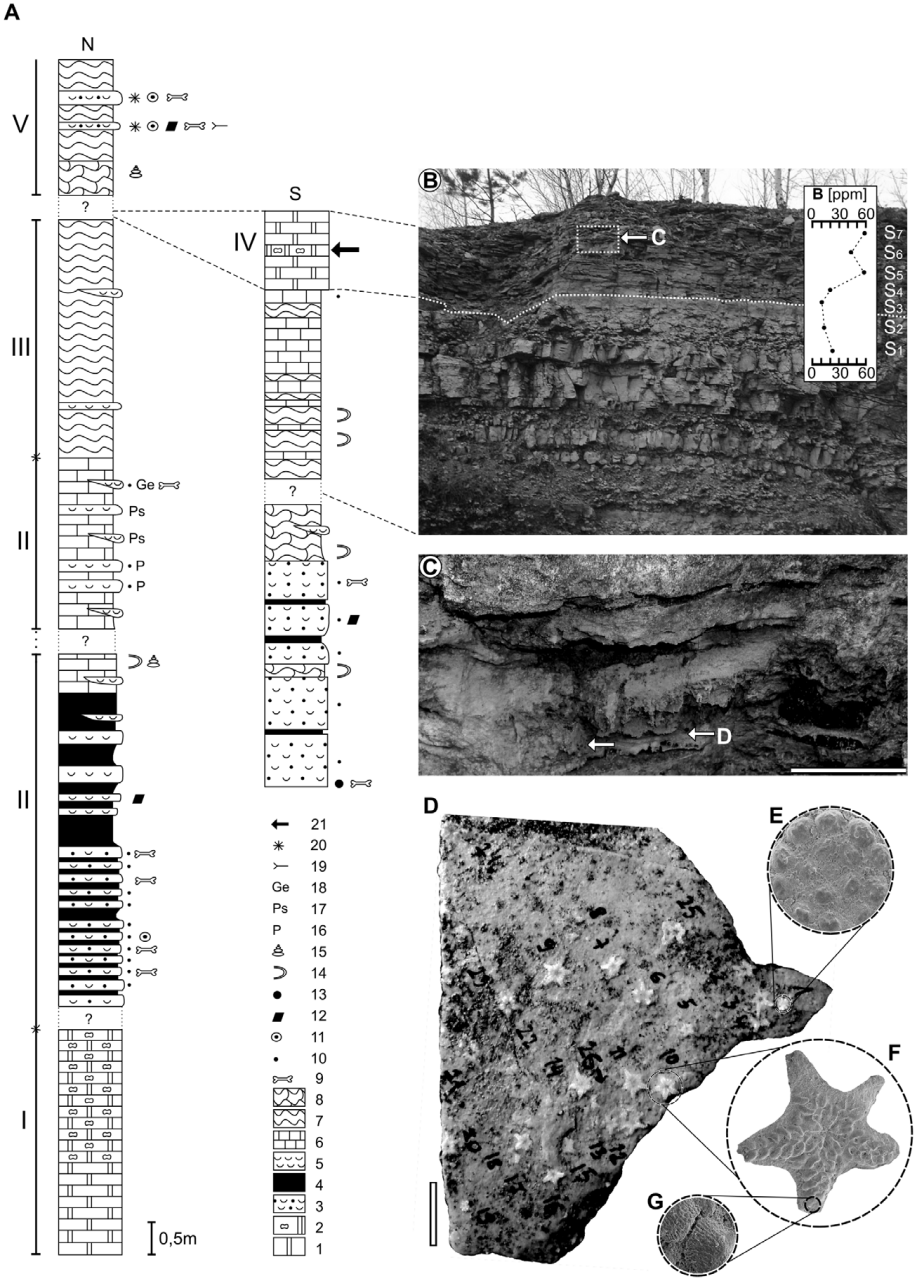
Stratigraphical section of Triassic sediments with ophiuroids. (**A**) Section of the northern and southern part of the Wojkowice Quarry (from [25]). 1, dolomitic limestones and marls; 2, cellular dolomitic limestones; 3, organodetrital limestones with bivalve detritus and columnals; 4, marly limestones; 5, pelitic limestones with abundance shells of bivalves; 6, pelitic limestones; 7, wavy limestones; 8, nodular limestones; 9, vertebrate remains; 10, *Dadocrinus* columnals; 11, encrinid columnals; 12, intraclasts; 13, regurgitalites; 14, *Rhizocorallium commune*; 15, numerous gastropods; 16, numerous *Plagiostoma*; 17, numerous *Pseudocorbula* sp.; 18, numerous *Gervillia* sp.; 19, *Thalassinoides*; 20, *Holocrinus* columnals; 21, layer(s) with presently recorded ophiuroids. I, Roetian; II, “limestones with *Entolium* and *Dadocrinus* unit”; III, “first wavy limestones unit”; IV, “cellular limestones unit”; V, “thick-bedded limestones” and “wavy limestones unit”. (**B**) Investigated section of “first wavy limestones unit” and “cellular limestones unit”; S_1_–S_7_ = rock samples for boron content analyses. Scale bar equals 1 m. (**C**) Enlargement of the ophiuroid layer. Arrows show the place of accumulations. Scale bar = 10 cm. (**D**) Slab (described in the paper as no. 1) with ophiuroid accumulation. (**E**) SEM micrographs of the oral view of the ophiuroid disc. (**F**) SEM micrographs of the aboral view of the near complete ophiuroid specimen. (**G**) SEM micrographs of the contact of arm plates showing relicts of the stereom microstructure. Scale bar = 10 mm.

In Wojkowice, carbonate deposits of the Upper Buntsandstein (Röt or Roetian) as well as the lowermost part of the Lower Muschelkalk (the Lower Gogolin Beds and lowermost part of the Upper Gogolin Beds; lithostratigraphic division of the Lower Muschelkalk after [24]) are exposed (detailed description of the lithostratigraphy and paleontology of the Wojkowice Quarry see [25]; Figure 2A). Two ophiuroid accumulations were found in the Cellular Limestones Unit (the so-called “Zellenkalk 2″) - uppermost lithological unit of the Lower Gogolin Beds. Its thickness in Wojkowice is up to ca. 1.3 m. This unit represents Aegean [26]. The Cellular Limestones Unit is considered equivalent of the Grenzgelbkalk and Liegende Dolomite in Germany [23], [27] and crops out in the whole area in the Upper Silesia where the sediments of Lower Muschelkalk are present. The thickness of these sediments varies from 0.8 to 2.0 m depending on the locality [24]. In contrast to other units of the Gogolin Beds, body fossils are extremely rare in the Cellular Limestones Unit of Silesia. Only occasionally, bivalves: *Hoernesia socialis* (Schlotheim), *Gervilleia mytiloides* (Schlotheim), *Myoconcha gastrochaena* Dunker and isolated crinoid ossicles were recorded [24]. In numerous outcrops of this unit, body fossils are absent [28], [29]. Similarly, ichnofossils were not documented whereas they are numerous in the Lower Muschelkalk (e.g., [23]).

In Wojkowice, the Cellular Limestones Unit is represented by yellow dolomitic limestones and dolomitic marls. Lower part of this unit consists of dolomitic limestones. Above, a few (45 cm thick) layers are exposed [23]: palisade calcite layer, dolocrete layer, rauhwacke, cellular limestones and rauhwacke layer, respectively. In the upper part of the Cellular Limestones Unit, dolomitic limestones and marls are exposed. Two dense accumulations of ophiuroids have been found on the upper surface of the cellular limestone layer that was covered by a thin (ca. 2 mm) muddy layer.

### Sampling

During the field work about 28 square metres of the sediments of the Celullar Limestones Unit (including 4 square meters layer of cellular dolomitic limestone) were investigated (Figure 2). Fossils are extremely rare in this unit: we recorded only three poorly preserved bivalve molds and surprisingly two accumulations of ophiuroids (26 and 6 specimens) that were found within one horizon near the upper surface of the cellular dolomitic limestone layer (Figure 2C,D). Both accumulations were separated from each other by a distance of ca. 80 cm. Additionally, three bulk samples from the cellular dolomitic limestone (each ca. 2 kg) were taken and transported to the laboratory of the Department of Earth Sciences of University of Silesia. No specific permissions were required for collection of fossils form this location. Field studies were carried out at an abandoned quarry with public right-of-way and did not involve endangered or protected species.

Slabs with ophiuroids were initially cleaned with hot water. Later the samples were slightly treated using peroxide in order to clean them off from the remnants of the thin layer of muddy sediments and again washed with hot water. Slabs were dried and watched under a binocular microscope for taphonomic studies. All ophiuroids from the Celullar Limestones Unit were examined carefully for evidence of breakage, abrasion, dissolution, regeneration traces and evidence of bite marks. Three selected bulk samples of dolomitic limestone were dissolved using glauber salt (8 cycles of boiling-freezing procedure). Later they were washed using hot tap water and sieved using Ø 0.5 mm, 0.315 mm and 0.1 mm mesh widths. After drying in 180°C, samples were screened under a binocular microscope.

### Petrographic and Geochemical Analyses

Polished and carbon-coated thin sections of the samples from the Celullar Limestones Unit were examined with cathodoluminescence microscope equipped with a hot cathode integrated with spectograph and linked to a Kappa video camera for recording digital images, at the Institute of Paleobiology of the Polish Academy of Sciences in Warsaw. Integration times for CL-emission spectra of luminescent samples were 50 s. The cathodoluminescent properties of sections were additionally examined using the Cambridge Image Technology Ltd CCL 8200 mk3 model system with 12–15 kV beam potential and 400 mA beam current attached to a NIKON ECLIPSE E400 Pol optical microscope at the Department of Stratigraphical Geology of Wrocław University. The petrographical investigations were carried out at the Institute of Geological Sciences of Wrocław University and at the Institute of Paleobiology of the Polish Academy of Sciences in Warsaw.

Seven samples (two from limestones and five from dolomitic limestones) were selected for geochemical analysis (in particular boron content) using PGNAA (Pulsed Gamma Neutron Activation Analysis) method (Figure 2B). 1 g samples are encapsulated in a polyethylene vial and placed in a thermalized beam of neutrons produced from a nuclear reactor. Samples are measured for the doppler broadened prompt gamma ray at 478 KeV using a high purity GE detector. Samples are compared to certified reference materials used to calibrate the system. A minimum of four standards are analyzed with every work order. Duplicates were analyzed to check method stability. The detection limit (0.5 ppm) reported is a function of the counting times required for each.

The collections are housed at the Department of Palaeontology and Biostratigraphy of the University of Silesia, Sosnowiec, Poland (catalogue number GIUS 7-3601– Geological Institute of the University of Silesia).

## Results

### Paleontology

Macerated samples did not contain any macro- or microfauna. However, surprisingly two dense ophiuroid accumulations were found in the field on the surface of two slabs. These ophiuroids were scattered across the area of 15 and 30 cm^2^, respectively. The first accumulation yielded 26 specimens whereas the second yielded only 6 specimens. All specimens were represented by complete or nearly complete forms (disc plus arms or partly preserved arms; see details below). Only 6% of total number of ophiuroids (2 specimens) were preserved *in situ*, i.e., with oral side directing towards the bottom (more details in discussion).

Ophiuroid specimens lack clear evidences of abrasion, extensive dissolution, regeneration or bite mark traces (Figure 2E,F,G). Three specimens yield (likely post-diagenetic) evidence of breakage (specimens no. 10 and 23 on the slab no. 1; specimens no. 2 on the slab no. 2; see Table 1,2). Most speciemens possess complete disc with partly preserved arms, i.e., 70% of speciemens lack distalmost part of arms. Two states of preservations can be distinguished:

1. Well-preserved ophiuroids (Taphonomic Group 1), with only minor signs of disarticulation (e.g., lack of distal arm portions);
2. More disarticulated specimens (Taphonomic Group 2) comprising central disc with attached less than five and partly preserved arms (having only proximal and median portions of their arms).

**Table 1:** Taphonomic features of *Aspiduriella similis* from Celullar Limestones Unit (accumulation no 1).

Specimen number	Disc diameter [in mm]	Life position: oral side up [u], down [d]	Breakage		Taphonomic group	
1	2.7	d		no	1	
2	2.9	u		no	1	
3	3.0	u		no	2	
4	3.0	u		no	2	
5	3.1	u		no	2	
6	2.8	u		no	2	
7	3.2	u		no	2	
8	3.3	u		no	2	
9	3.2	u		no	2	
10	2.9	u		yes	2	
11	3.0	u		no	2	
12	2.9	u		no	2	
13	3.4	u		no	1	
14	3.1	u		no	2	
15	3.2	u		no	2	
16	2.8	u		no	2	
17	2.8	u		no	2	
18	2.9	u		no	2	
19	3.1	d		no	1	
20	3.3	u		no	1	
21	3.2	u		no	1	
22	3.1	u		no	2	
23	3.0	u		yes	1	
24	2.8	u		no	1	
25	3.0	u		no	2	
26	3.1	u		no	1	

**Table 2:** Taphonomic features of *Aspiduriella similis* from Celullar Limestones Unit (accumulation no 2).

Specimen number	Disc diameter [in mm]	Life position: oral side up [u], down [d]	Breakage		Taphonomic group	
1	3.0	u		no	2	
2	2.8	u		yes	2	
3	2.7	u		no	1	
4	2.8	u		no	2	
5	2.9	u		no	2	
6	3.0	u		no	2	

### *Aspiduriella* Description

The ophiuroid assemblages from Wojkowice consist of monospecific and multiindividual accumulation with small ophiuroids *Aspiduriella similis* (Eck) with narrow range size 2.7–3.4 mm (Table 1,2). This species is thought to have been a slow-moving, low-level epifaunal detritivore and/or suspension feeder [30]. These ophiuroids have central disc of oval outline (Figure 2E). The center of the disc has a small and distinctly pentagonal centrodorsal plate. The first circlet of disc is composed of five hexagonal basal plates. The radial plates are relatively large and drop-shaped; they are covered by single and small protuberances on the radial position on each plate. Numerous and irregular secondary plates occur between basal and radial plates. The arms are rather short and their tips are usually not preserved. The lateral plates in the proximal part are narrow and low. The dorsal plates are smooth, romboid and decrease in size distally (Figure 2F). The ventral plates have sharp edges.

### Petrography and Geochemistry

**Figure 3:**
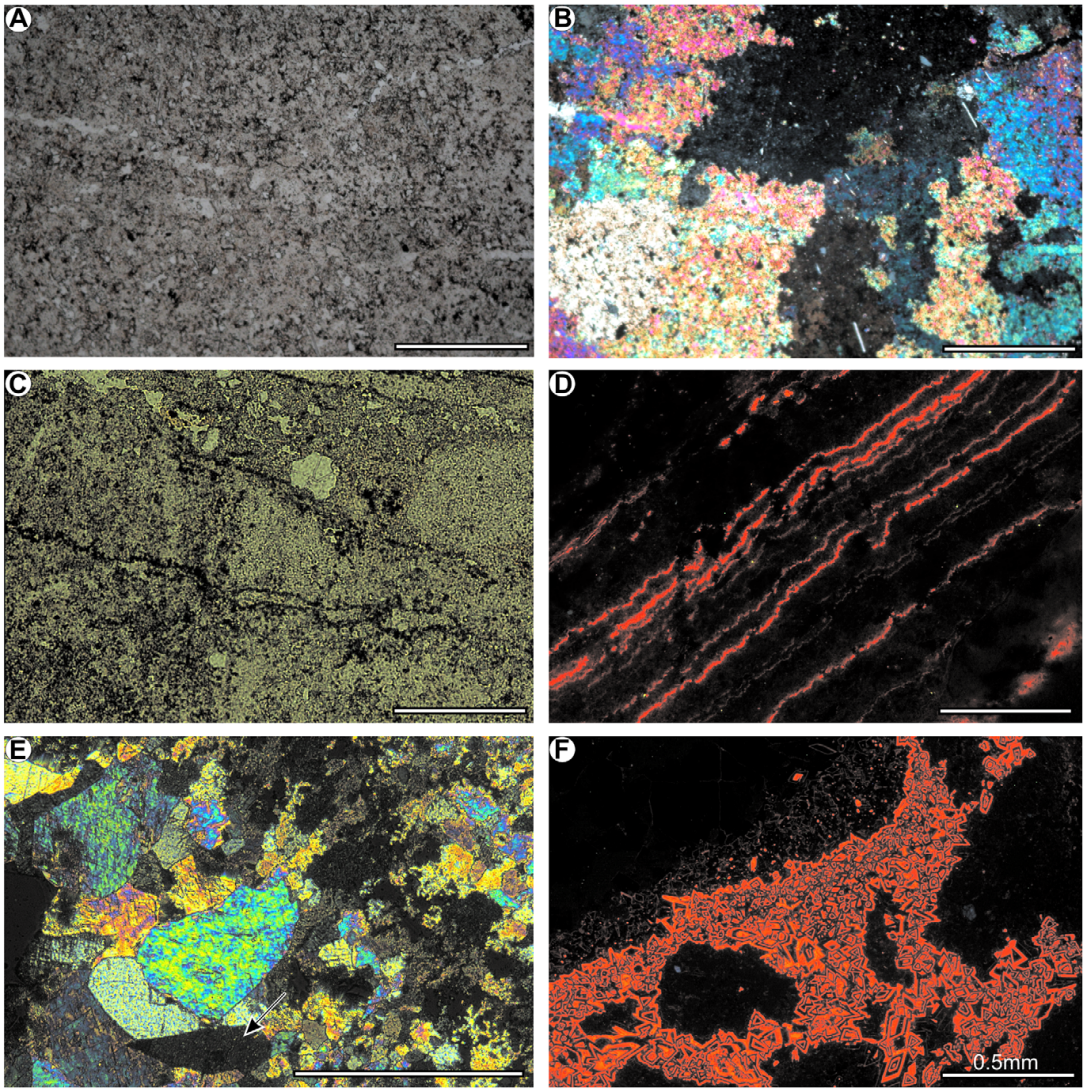
Petrographical and cathodoluminescent features of the layer with ophiuroids. (**A**) Fine-grained dedolomites under transmitted light and (**B**) in crossed-nicoles. (**C**) Laminated dedolomites with irregulary distributed voids under transmitted light. (**D**) Laminated and microfolded dedolomites under cathodoluminescence. (**E**) Pseudospar crystals and rhombohedral crystals of calcitized dolomites and pseudomorphs after gypsum (arrows) in crossed-nicoles. (**F**) Rhombohedral crystals of calcitized dolomites under cathodoluminescence showing zoned crystal growth. Dolomite rhomboids have non-luminescent and bright orange luminescent zones. Non-luminescent areas are due to Fe rich diagenetic fluids and the incorporation of Fe in the dolomite lattice (“quench ion”) during crystal gwowth, bright orange luminescent areas indicates changes in the diagenetic fluid chemistry into Mn enrichment and the incorporation of Mn in the dolomite lattice (“activator ion”) during crystal growth.

**Figure 4:**
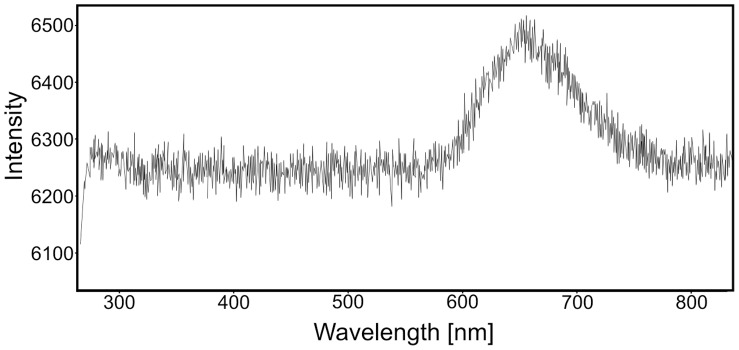
CL-activated UV-VIS spectrum of the luminescent dolomite (see **Figure 3F** **).** Spectrum shows Mn^2+^ emission maximum at ca. 660 nm (Mn^2+^ activation in the MgCO_3_ position).

The cellular limestone layer consists of fine-grained, sometimes laminated and microfolded dedolomites with irregulary distributed voids built mainly of fine pseudospar crystals (Figure 3). Occasionally, pseudomorphs after gypsum (Figure 3E) and rhombohedral crystals of calcitized dolomite are observed. Some of them possess distinct zonal structures (Figure 3F). The dedolomites contain Fe-Mn oxides and hydrooxides as suggested by cathodoluminescent analyses, i.e., non-luminescent zones are due to Fe^2+^-rich (“quencher ion”) diagenetic fluids whereas bright orange luminescent areas are due to changing diagenetic fluid chemistry leading to Mn^2+^incorporation (“activator ion”). The CL emission spectrum of an orange luminescing Mn^2+^-activated showed emission maximum at about 660 nm (Figure 4, Mn^2+^activation in the MgCO_3_ position).

Geochemical analyses revealed that the boron is non-uniformly distributed within a part of investigated section. The main feature of boron distribution is a relatively low B concentration in the lower part of the section (10–23 ppm) and much higher concentration in upper part of the section (44–58 ppm) (see Figure 2B).

## Discussion

### Depositional Paleoenvironment of Cellular Limestones Unit

The Silesian Cellular Limestones Unit (Zellenkalk 2) was formed at the end of the highstand system tracts phase, when significant shallowing of the basin occurred [23]. This led to the temporarily emergence of the bottom and strong evaporation which is supported by isotopic data of δ^13^C and δ^18^O [23], [31]. The Cellular Limestones Unit has no evidence of marine intercalations and was presumably deposited in sabkha environment with a dominant sedimentation of lime mud [31]. Analogical cellular structures (cells) in cellular limestone in Silesian Roetian (Zellenkalk 1) are interpreted as voids after dissolved and removed gypsum and halite crystals [32]. Similar voids from Zellenkalk 2 of the same origin are observed (Figure 3 C).

Supra-normal salinity during deposition of this unit is supported by low abundance and diversity of fossils (3 bivalve molds and two opiuroid accumulations on 28 square metres investigated) and lack of ichnofossils. By contrast, fossils (especially crinoids) are numerous in the adjacent lithological units [25], [33]. It is noteworthy that previous studies have documented three bivalve species within the Cellular Limestones Unit: *Hoernesia socialis* (Schlotheim), *Gervilleia mytiloides* (Schlotheim), *Myoconcha gastrochaena* Dunker [24]. According to the literature [34], all these species are euryhaline. Especially, *Hoernesia* has been commonly reported in hypersaline deposits of Europe and Canada [34], [35].

The occurrence of some postevaporatic structures (including layer of palisade calcite and the rauhwacke layers) is also consistent with increased salinity interpretation [23] although according to some authors [31] the Cellular Limestones Unit comprises also early diagenetic evaporites. Additionally, our microfacial observations indicate that within the Cellular Limestones Unit two standard microfacies types can be distinguished: SMF 23: Non-laminated homogeneous micrite and microsparite without fossils (Figure 3A,C) and SMF 25: Laminated evaporite-carbonate mudstone (Figure 3D) which are both indicative of increased salinity (such as sabkha) environment [36]. It is worth mentioning here that an equivalent of the Silesian Cellular Limestones Unit in Thuringian Basin, the so-called Gelbkalke is also interpreted as saline dolomite formed in sabkha environment [37].

It has been argued that light stable isotopes (δ^13^C and δ^18^O) may be useful in paleosalinity reconstruction [38]. However, our petrographical data suggests that lime mud in Wojkowice underwent early diagenesis and later complete calcitization. Therefore, any isotopic analyses within this layer are herein impossible. Nevertheless, another important argument for an increased salinity comes from geochemical data. It has been suggested that boron content of the sediment shows a positive correlation with salinity and can be used as paleosalinity indicator [39]–[47]. However, many complexities (including diagenetic changes, different illite and organic carbon concentrations) can plague the use of boron as a paleosalinity proxy [48]. Nevertheless, other workers indicated a lack of correlation between boron concentration and either the clay-silt ratio or the percent organic carbon [47] as well as degree of diagenetic changes [49]. Results of our geochemical analyses indicate a remarkable boron concentration (up to about 58 ppm) in the Cellular Limestones Unit (Figure 2B). By contrast, boron concentration in the lower part of the section (considered fully marine) is more than twice lower.

### Origin and Taphonomy of Ophiuroid Accumulations from Wojkowice

Considering the origin of ophiuroid accumulations described here, it is necessary to determine if these accumulations are autochthonous or allochthonous (e.g., transported from the nearshore part of the marine basin onto the sabkha by storms). Layer with ophiuroids, similarly to the adjoining layers, consists of fine-grained (mud fraction) sediment without any sedimentary structures which are indicative of high energy environment (e.g., intraclasts, channel erosion, ripple marks, grading). The top surface of the layer with ophiuroids is plain with no signs of erosion and is covered by an undeformed thin (ca. 2 mm) muddy layer. The influence of incidental tidal sedimentation on the exposed and dried surface is also unlikely as such deposits typically yield characteristic structures (e.g. mudcracks, raindrops or herringbone cross-stratification) that are not seen within the investigated unit. Furthermore, transportation induces characteristic signs of abrasion on the ossicle surface which are not observed in studied ophiuroids (Figure 2E,F,G). Only 3 specimens yield evidence of (possibly post-diagenetic) breakage. If the sediment with ophiuroids was transported by storm, Cellular Limestones Unit should contain other macro- and microfossils (such as crinoids, foraminifers, ostracods) that are commonly present in marine deposits of the Gogolin Beds. However, in analysed samples macro- and microfossils were not documented (except bivalve molds). Admittedly, although lack of the distalmost parts of the arms in 70% of the specimens may be indicative of transport, it has been argued that storms do not appear to cause arm damage in extant living ophiuroids [50]. A possible explanation for that mode of preservation is that after the death, initially distalmost parts of the arms become disarticulated and as the smallest and the lightest ossicles they could have been easily transported. It cannot be completely excluded that some of the arms could have been also autotomized before death.

The found ophiuroids are generally classified within the first taphonomic group (Type 1 echinoderms; [51]). This group comprises echinoderms with ossicles that are held together only by soft tissues such as ligaments and muscles. These echinoderms do not remain articulated for very long after death because their ossicles are rapidly disarticulated by decay [51]. Therefore, the perfect state of preservation of our ophiuroids indicates rather short post-mortem seafloor exposure. Taken together, it seems that these ophiuroids appear to be para-autochthonous in that they have not been transported away from their life habitat.

Extant echinoderms living in hypersaline environments commonly display dwarfism [22], [52], [53]. Studied ophiuroids from Wojkowice are relatively small (max. disc diameter equals 3.4 mm, mean disc diameter equals 3.0; see Table 1,2). However, data on size range as well as relationship between the size and the age of the specimens of Triassic *Aspiduriella* are rather sparse. Within the genus *Aspiduriella* several species have been documented: *A*. *camuna* (Rossi Ronchetti), *A*. *dorae* (Lepsius), *A*. *italica* (Crema), *A*. *ludeni* (von Hagenow), *A*. *montserratensis* (Calzada and Gutiérrez), *A*. *scutellata* (Blumenbach), *A*. *similis* (Eck) and *A*. *streichani* (Kutscher M.) [54]–[58]. However, of these species only four are typical for the Muschelkalk Germanic Basin (*A*. *ludeni*, *A*. *scutellata*, *A*. *similis* and *A*. *streicheni*). The most reliable size data is the maximum disc diameter. *A*. *ludeni* occurring commonly in the Lower Muschelkalk of the Polish part of the Germanic Basin is the largest form with disks reaching up to 8.2 mm. This form was also noted from the Lower Muschelkalk of the eastern Germany (Rüdersdorf near Berlin) [57], [59]–[61]. *A*. *streichani* is similarly-sized to *A*. *ludeni* and its disk reached up to ca. 8 mm. This species is common only in the vicinity of the Lower Muschelkalk of Rüdersdorf [62]. However, it should be pointed out that the morphology of this species is very similar to the previously mentioned *A*. *ludeni*. Stoll [63] ascribed this taxon to *A*. *ludeni* with a question mark. *A*. *scutellata* with its maximum disk diameter equaling 6.9 mm occurs in the Lower and Upper Muschelkalk of Poland and Germany [54], [55], [62]–[64]. This species is the only one that also has been reported from the Tethyan Realm of Italy [65]. The maximum disk diameter of *A*. *similis* commonly occurring in the Lower Muschelkalk of Poland equals 5 mm [66]. Unfortunately, data on range size as well as mean size are sparse. Previous workers [30], [66] have mentioned that ophiuroids from various localities in Poland have mean disc diameter commonly not exceeding 3 mm. However, these ophiuroid taphofacies are connected with transportation from their life habitat due to storm-related obrution events. In such cases, segregation of the skeletons is likely. Thus, data on mean disc diameter of these ophiuroid populations are probably underestimated. Therefore, given our current state of knowledge on ontogenesis of *A*. *similis* it is impossible to clearly state if ophiuroids from Wojkowice underwent dwarfism or not.

Good state of preservation (see taphonomic groups in Table 1,2) and an overall sedimentological context indicate that the death of ophiuroids was not caused by high-energy (storm or tidal) event. Predation is also not a likely cause of their death as ophiuroids do not display evidence of damages of their discs and arms (such as bite marks comparable to those observed in crinoids from the lower part of this section; [33], [67]). Furthermore, the Cellular Limestone Unit in the Wojkowice lacks potential body predatory fossils (e.g., cidaroids, crabs, hybodontid sharks) or ichnofossils (e.g., *Thalassinoides*, *Rhizocorallium*) that are ascribed to predatory decapods. Such fossils do not occur also in other outcrops of this unit in the Upper Silesia [23], [24], [68], [69].

One possible explanation is that the ophiuroids from Wojkowice died due to the gradual deterioration of environmntal conditions. In the unstable sabkha environment, progressive evaporation might have been stressful for ophiuroids. For example, subaerially weathered dolomites indicate that this area might have been episodically lifted up [23]. In such drying sabkha, elevated temperatures, increasing salinity, as well as progressive oxygen depletion might have been responsible for the ophiuroid death. The fact that nearly all ophiuroids are preserved with their oral side turned up is consistent with such interpretation as studies on modern ophiuroids from the Northern Adriatic Sea [70] indicate that oxygen depletion commonly leads to their arm-tipping and the accompanying uplifted disc [71]–[72]. This behaviour is generally interpreted as an attempt to reach higher oxygen concentrations. In such humped postures, ophiuroids are prone to overturn. Similar arm-tipping behaviour have been recorded elsewhere during hypoxia in modern opiuroid species, including *Ophiura texturata* (Linnaeus) [73], [74], *O*. *albida* (Forbes) [75], or *Amphiura chiajei* (Forbes) and *A*. *filiformis* (Müller) [76], [77]. Alternatively, other factors may have also contributed to such an inverted position. It is noteworthy that extant ophiuroids can change body posture to an upside down position, with oral side of the disc facing upward and arms raised above the disc [78]. Furthermore, these organisms can curl into a ball-shaped configuration leading to the overturn due to shift of the center of gravity. Finally, the inverted position of extant ophiroids has been also reported in ophiuroids escaping from sediments [79]. After the overturn of ophiuroids, initially distalmost parts of the arms became disarticulated (if not earlier autotomized) and as the smallest and the lightest ossicles could have been easily transported. Then, within several? days the speciemens must have been covered by thin ca. 2 mm thick layer of the sediment allowing their near-complete preservation [51].

### Conclusions

Fossil echinoderms are generally considered ideal indicators of fully marine conditions. Thus they have been commonly used in paleoenvironmental reconstructions. For example, echinoderm fossils (in particular asterozoan traces) have recently warranted a re- interpretation of the depositional environment of Stuttgart Formation (Middle Keuper) from the non-open marine into fully marine environment [80]. Discovery of ophiuroid accumulations from the Middle Triassic of Poland constitutes the first and the oldest fossil record to our knowledge of echinoderms found in hypersaline environment. Our discovery suggests that some echinoderm species might have been euryhaline and imply that adaptation to increased salinity might have already appeared in the Middle Triassic. Therefore, great care needs to be exercised when using fossil echinoderm as paleoenvironmental proxies.
