# Correction: Ophiuroids Discovered in the Middle Triassic Hypersaline Environment

**DOI:** 10.1371/annotation/925b10db-45ac-4d63-81e6-4e0cd034b7a2

**Published:** 2013-05-31

**Authors:** Mariusz A. Salamon, Robert Niedźwiedzki, Rafał Lach, Tomasz Brachaniec, Przemysław Gorzelak

There were three typographical errors in the second and third paragraphs of the Introduction. The values "7.7%", "55%", and "60%" are incorrect. The correct values are "7.7‰", "55‰", and "60‰". 

